# Bacterial and Fungal Community Shifts and Environmental Drivers in Talahao Coal Mine Water

**DOI:** 10.3390/microorganisms14092041

**Published:** 2026-09-13

**Authors:** Limin Yang, Youkun Huang, Duoen Gu, Yifei Gu, Jianyu Ma, Danyang Liu, Huiting Wu, Zhuqing Feng, Zhaowen Liu, Junyi Wang

**Affiliations:** 1 School of Materials and Environmental Engineering, Chizhou University, Chizhou 247000, China; 2 Anhui Provincial Key Laboratory for Quality and Safety of Agri-Products, School of Resource and Environment, Anhui Agricultural University, Hefei 230036, China

**Keywords:** Talahao coal mining, bacterial diversity, fungal diversity, environmental factors, ecological restoration

## Abstract

Underground coal extraction alters aquatic ecosystems. Quantitative evaluation of microbial responses serves as an indicator for ecological security. High-throughput amplicon sequencing and structural equation modeling were applied to analyze aquatic bacterial and fungal communities before and after operations at the Talahao coal mine. Mining activities correlated with altered physicochemical water properties and reduced microbial community evenness. Environmental variables, including ion accumulation and suspended solid concentrations, mediated the indirect effects of mining on microbial restructuring based on structural equation models. Taxonomic composition shifted across domains following extraction. Pre-mining bacterial communities contained higher proportions of the genera *Devosia* and *Acidovorax*. Post-mining bacterial communities experienced an enrichment of the genus *Malikia* and the family Methylomonadaceae. Pre-mining fungal communities included *Cladosporium* and *Aspergillus*. Post-mining fungal communities exhibited a subterranean response pattern dominated by unclassified lineages and taxa belonging to Basidiobolaceae diverging from surface soil reference frameworks. Functional predictions indicated divergent stability between the two domains. Bacterial inferred functional profiles remained stable despite taxonomic restructuring. Fungal assemblages underwent compositional shifts in trophic guilds. These data provide a baseline regarding the divergent functional stability between bacterial and fungal communities in water bodies before and after underground coal extraction and establish biological indicators for environmental assessment.

## 1. Introduction

Coal extraction supports global economic growth [1], alongside environmental degradation such as land subsidence, soil pollution, and microecological disruption [2]. These conditions alter native soil flora, fauna, and microbial communities [3,4]. Subsidence from mining converts terrestrial habitats into waterlogged lake ecosystems and reduces surface water quality [5]. Landscape structural changes disrupt soil pore distribution and nutrient retention, limiting plant growth and microbial stability [6]. The transformation of collapsed soil into sediment alters organic matter decomposition and reduces microbial diversity [7]. Underground mineral extraction disrupts deep groundwater aquifers, increasing pollutant diffusion and restructuring microbial communities along depth gradients [8]. Soil and aquatic microbiomes regulate nutrient cycling and ecosystem processes [9]. Changes in their biomass, diversity, and taxonomic composition due to mining impact ecosystem function [10]. Evaluating microbial community responses to pollution from mining provides a basis for comprehensive environmental assessment.

Ecological changes in coal mining areas are evaluated through microbial diversity sequencing [11]. Analysis of community structure informs the functional microorganism screening and remediation strategy optimization for environmental governance [12]. This sequencing method details the composition and diversity of microbial communities in extreme environments such as soil, tailings, and collapsed lake sediments in mining areas at high resolution without cultivation [13]. This method provides biological indicators for evaluating ecological disturbances caused by coal extraction. Comparing community differences across pollution gradients identifies groups resistant to acid and heavy metals, revealing microbial adaptation mechanisms to acidic mine drainage and substrates with high sulfur and heavy metals [14]. Data from 16S rRNA sequencing characterize microbial geographic patterns between collapsed lakes and detail the driving effects of chemical factors, including pH, total nitrogen, and total phosphorus on benthic bacterial communities [15]. This approach further identifies functional bacteria involved in sulfur, phosphorus, and organic matter cycling, showing how sediment bacteria drive phosphorus fixation and heavy metal precipitation through metabolic pathways to alleviate pollution diffusion [16]. Internal transcribed spacer sequencing provides data with high resolution regarding fungal community dynamics within disturbed ecosystems. Fungi are decomposers and potential pathogens in mine waters [17]. These eukaryotic assemblages display lower taxonomic diversity than bacterial counterparts yet mediate organic matter decomposition and structural nutrient cycling. These microorganisms exhibit sensitivity to hydrochemical fluctuations. Quantifying shifts in saprotrophic and pathogenic fungal guilds generates biological indicators for evaluating ecosystem resilience following mining degradation. Sequencing data reveal an increasing trend of Proteobacteria and enhanced activity of rare operational taxonomic units with increasing aquifer depth [18]. This trend functions as a biological indicator of aquifer pollution connectivity. Integrating functional prediction tools facilitates the inference of changes in bacterial phenotypes and fungal niches, supporting the screening of tolerant strains for bioremediation [12].

Water samples were collected at the Talahao coal mine to analyze microbial community characteristics before and after mining (AM) activities. The site is located at the junction of Tongchuan Town, Dongsheng District, Ordos City, Inner Mongolia Autonomous Region, and Zhungeerzhao Town, Zhungeerqi. Mine construction began in September 2012. Operations commenced in July 2016, and production transition occurred by February 2020. Underground mining caused overlying strata movement and destruction one year after operations began. Early extraction formed a goaf area of 11.8219 km^2^ by March 2024, triggering a ground subsidence area of 12.9722 km^2^. Ecological restoration across an 11 km^2^ area required an investment exceeding 160 million yuan and received expert approval in December 2024. Environmental degradation persists at the Talahao coal mine. This ongoing degradation indicates a requirement for optimizing current remediation strategies. Analyzing geological conditions and microbial communities provides a basis for environmental remediation.

Internal transcribed spacer sequencing provides high-resolution data on fungal community dynamics within disturbed ecosystems. Fungi are decomposers and potential pathogens in mine waters. These microorganisms exhibit sensitivity to hydrochemical fluctuations. Quantifying shifts in saprotrophic and pathogenic fungal guilds establishes biological indicators for evaluating ecosystem resilience following mining degradation. High-throughput amplicon sequencing and structural equation modeling were applied to analyze bacterial and fungal communities in Talahao coal mine water before and after operations. Objectives included quantifying alterations in microbial diversity, identifying physicochemical variables governing community assembly, and determining functional responses of bacteria and fungi to mining impacts. These data offer empirical evidence of environmental variables mediating microbial community restructuring. The integrated approach establishes quantitative biological indicators for evaluating environmental disturbances and informs ecological restoration strategies in coal mining ecosystems.

## 2. Materials and Methods

### 2.1. Sample Collection and Analysis

**Figure 1:**
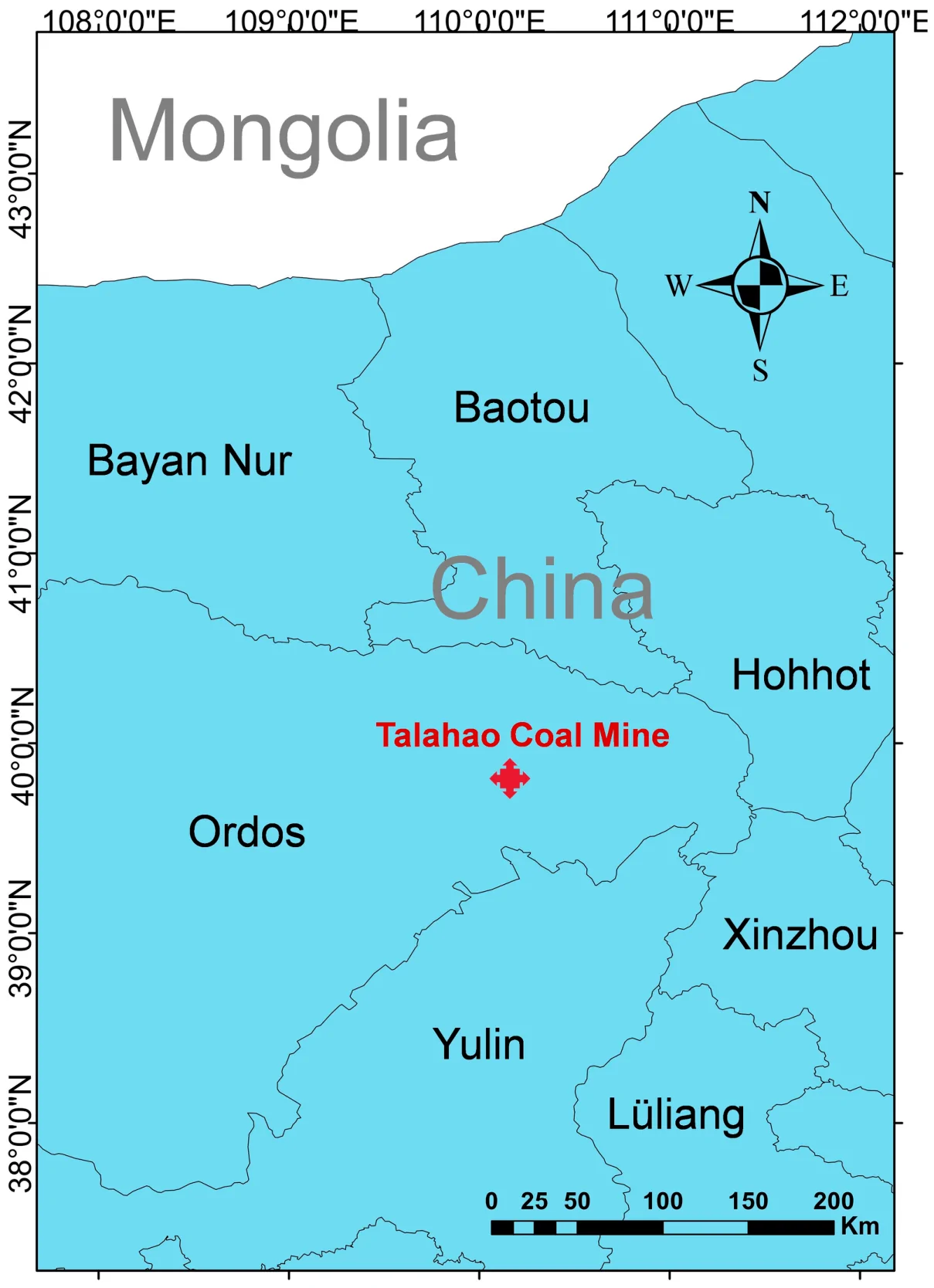
Location map of the study area showing the Talahao Coal Mine in Ordos City, Inner Mongolia Autonomous Region, China.

Microbial community dynamics before and AM activities were evaluated using a composite sampling strategy at the Talahao coal mine (110°08′25″ E, 39°45′55″ N). The site is located at the junction of Tongchuan Town in Dongsheng District, Ordos City, Inner Mongolia Autonomous Region and Zhungeerzhao Town in Zhungeerqi (Figure 1). All sampling points were situated within the active mining area of the third coal seam (out of six total seams) at a depth of 208 m beneath the surface. Before Mining (BM) samples were collected on 19 August 2024 from clean coal seam seepage water within established structural tunnels. These locations were unaffected by active coal extraction, which commenced on 26 August 2024. AM samples were collected from the active mining face after extraction began. These samples contained suspended coal dust, reflecting disturbed environmental conditions. Water samples were formulated by pooling liquid subsamples from six distinct locations at each point to overcome spatial heterogeneity, with three biological replicates taken per condition. Sterile 50 mL centrifuge tubes (Corning Incorporated, Corning, NY, USA) were utilized for collection. All samples were sealed immediately upon collection and preserved in liquid nitrogen and dry ice.

### 2.2. Determination of Environmental and Physicochemical Parameters

Physicochemical monitoring of the goaf environment (Table 1 and Table 2) was conducted concurrent with microbial sampling. Ambient gas concentrations, including methane (CH_4_), carbon dioxide (CO_2_), sulfur dioxide (SO_2_), and carbon monoxide (CO), were measured in situ utilizing a portable multi gas detector (RAE Systems, San Jose, CA, USA). Water quality parameters such as pH, temperature (Tm), and total dissolved solids (T-DS) were recorded at the 208 m depth using a portable multiparameter water analyzer (Hach Company, Loveland, CO, USA). Collected water samples were transported to the laboratory for chemical analysis. Suspended solids (SS) were measured via the standard gravimetric method. Chemical oxygen demand (COD) and five-day biochemical oxygen demand (BOD_5_), along with nutrient levels such as ammonia nitrogen (NH_3_-N) and total phosphorus (T-P), were quantified using spectrophotometry (UV-2600, Shimadzu Corporation, Kyoto, Japan). Concentrations of specific anions and cations, including sulfate (SO_4_^2−^), iron (Fe^2+^), and manganese (Mn^2+^), were determined using ion chromatography (CIC-D120, Qingdao Shenghan Chromatograph Technology Co., Ltd., Qingdao, China).

### 2.3. DNA Extraction, Amplicon Library Preparation, and 16S rRNA Gene/ITS Sequencing

Genomic DNA was extracted from filtered water samples utilizing the TaKaRa MiniBEST Universal Genomic DNA Extraction Kit Ver. 5.0 in accordance with manufacturer guidelines. Extracted DNA was processed through a column purification kit to eliminate metal ions and humic substances that inhibit polymerase chain reactions in mineralized mine water samples. DNA quality and concentration were evaluated utilizing a NanoDrop 2000 spectrophotometer (Thermo Fisher Scientific, USA). Amplification inhibition was assessed through preliminary amplification of serially diluted template DNA. All template DNA concentrations were standardized to 10 ng/μL prior to library preparation. Sterile water was incorporated as blank extraction controls and polymerase chain reaction negative controls to monitor environmental contamination. These controls were sequenced alongside experimental samples, and background contaminant sequences were computationally subtracted from the final operational taxonomic unit dataset. The V3 and V4 regions of the bacterial 16S rRNA gene were amplified using the primer pair 338F and 806R. The internal transcribed spacer region was targeted for fungal community analysis using the primer pair ITS1F and ITS2R. Amplicons were sequenced on the Illumina MiSeq platform (Majorbio Bio Pharm Technology, Shanghai, China). Raw sequencing reads underwent quality filtration using fastp and merging via FLASH. High-quality sequences were clustered into operational taxonomic units at 97% sequence similarity utilizing USEARCH, and chimeric sequences were discarded during the clustering step [19,20]. Taxonomic assignments for representative sequences were performed via the RDP Classifier against the SILVA database for bacterial sequences and the UNITE database for fungal sequences [21,22].

### 2.4. Statistical Analyses

Bioinformatic processing and statistical analyses were performed via the integrated pipelines of the Majorbio Cloud Platform. Data normality was assessed using the Shapiro–Wilk test. Significant differences in microbial diversity between the BM and AM groups were evaluated using the Student *t*-test for normally distributed data and the Wilcoxon rank-sum test for nonparametric data. *p*-values were adjusted for multiple testing using the False Discovery Rate (FDR) control. Beta diversity was visualized via Principal Coordinate Analysis (PCoA) based on Bray–Curtis dissimilarities, with statistical significance determined by PERMANOVA (Adonis). Bacterial phenotypic traits were predicted using BugBase based on the 16S rRNA OTU table, and fungal functional guilds were assigned using the FUNGuild database based on the ITS OTU table [23].

### 2.5. PLS-SEM Modeling of Bacterial and Fungal Communities

Causal pathways linking mining activities, environmental factors, and water physicochemical properties to microbial community characteristics were elucidated using structural equation modeling based on the partial least squares approach. Two independent partial least squares structural equation modeling (PLS-SEM) models were constructed for the bacterial and fungal communities to capture distinct response patterns. All analyses were performed using SmartPLS 4 (Version 4.0, SmartPLS GmbH, Germany). The PLS algorithm with a path weighting scheme was applied for model estimation, with a maximum of 500 iterations and a convergence threshold of 10^−7^. The significance of structural path coefficients was assessed via nonparametric bootstrap resampling (5000 subsamples), and 95% bias-corrected and accelerated (BCa) confidence intervals were computed. Statistical significance was declared at *p* < 0.05. The explanatory power of the endogenous latent variables was evaluated by the coefficient of determination (*R*^2^). Discriminant validity was verified by the Fornell–Larcker criterion and the heterotrait-monotrait ratio of correlations. Multicollinearity was diagnosed using the variance inflation factor (VIF), and overall model fit was appraised by the standardized root mean square residual (SRMR).

## 3. Results

### 3.1. Genus-Level Composition of Bacterial and Fungal Communities in Mine Water

**Figure 2:**
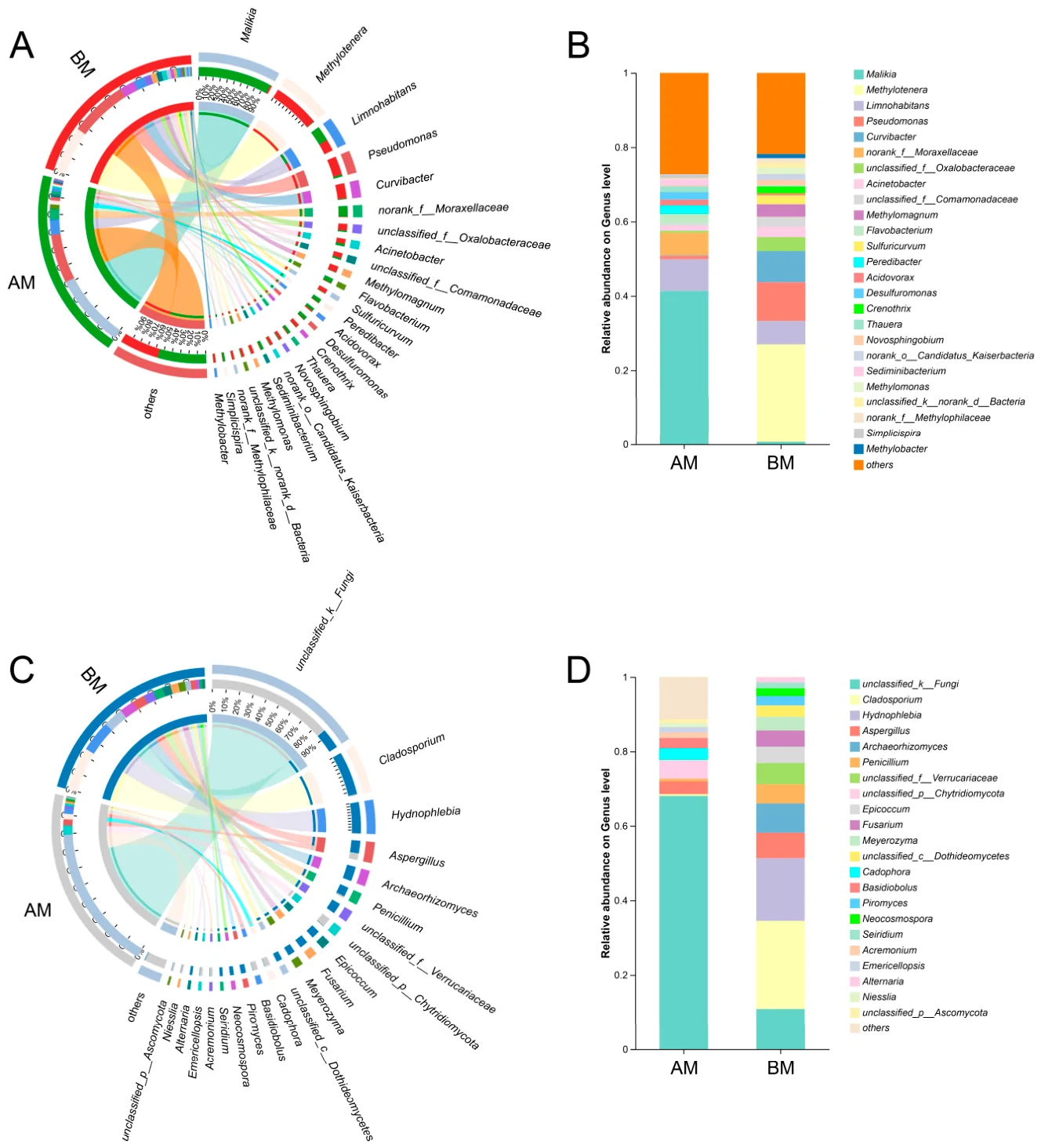
Composition and relative abundance of microbial communities at the genus level. (**A**) Circular diagram showing the bacterial community structure in AM and BM; (**B**) stacked bar chart of bacterial relative abundance; (**C**) circular diagram showing the fungal community structure in AM and BM; and (**D**) stacked bar chart of fungal relative abundance.

Differences in the relative compositions of bacterial and fungal communities between BM and AM water samples were identified via high-throughput sequencing (Figure 2). Taxonomic relative abundance distributions of bacterial communities were even prior to mining. Community structure differed in samples collected AM. The relative abundance of the genus *Malikia* constituted approximately 40 percent of the total bacterial sequences, compared to a near absence in pre-mining samples. Fungal communities showed a distinct compositional state following mining. Pre-mining water contained fungal taxa, including *Cladosporium* and *Aspergillus*. A single operational taxonomic unit accounted for approximately 70 percent of the total fungal sequences in post-mining samples. This sequence was unclassified at the genus level but assigned to the Kingdom Fungi. This dominant representative sequence was verified via BLAST + 2.17.0 against the NCBI database to exclude technical artifacts, chimeras, and non-fungal amplicons.

### 3.2. Hierarchical Clustering of Dominant Bacterial and Fungal Genera

**Figure 3:**
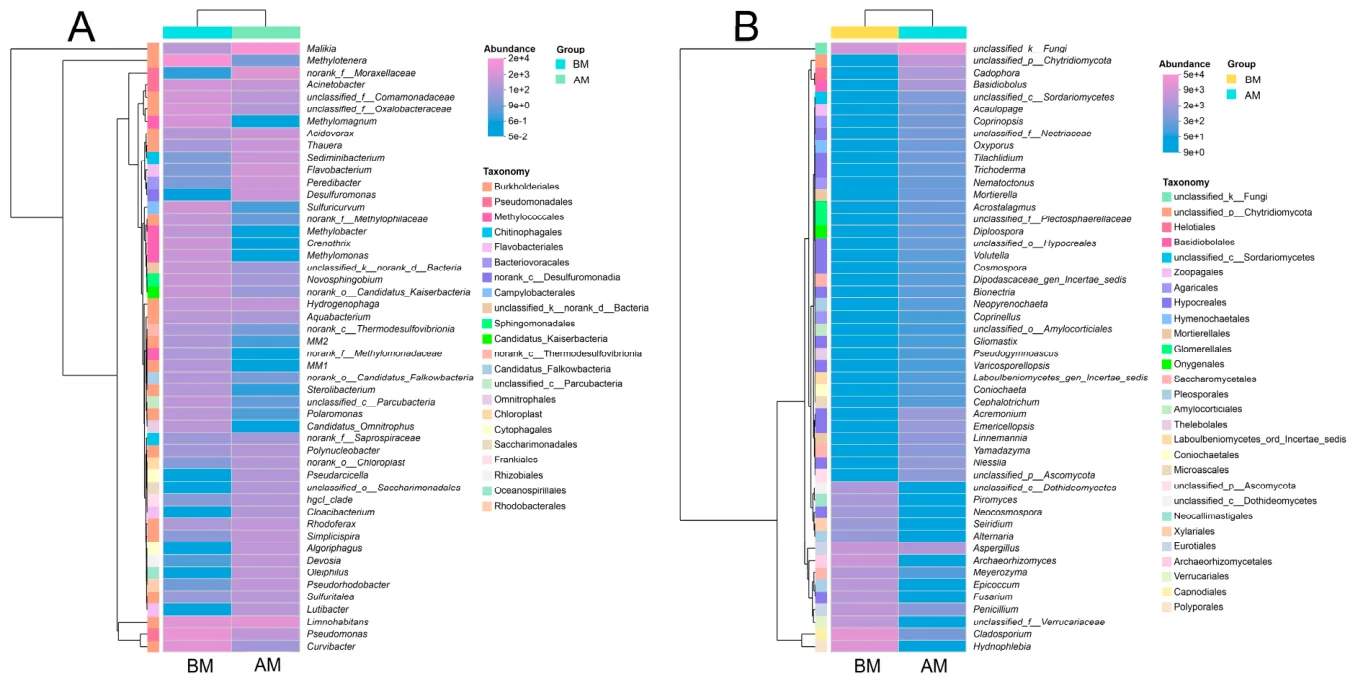
Heatmap analysis of differentially abundant bacterial (**A**) and fungal (**B**) taxa between AM and BM samples, with hierarchical clustering based on abundance patterns.

Differences in the relative abundances of microbial taxa between BM and AM water samples were visualized using hierarchical clustering heatmaps (Figure 3). Bacterial community samples clustered according to the sampling period (Figure 3A). Higher relative proportions of genera, including *Devosia* and *Acidovorax*, were observed in BM samples. AM samples were characterized by a statistically significant relative enrichment of *Malikia* (False Discovery Rate adjusted *p* < 0.05), and groups within the Methylomonadaceae family. Fungal communities clustered based on sampling period (Figure 3B). Fungal taxa, including *Cladosporium* and *Aspergillus*, were associated with BM conditions across all biological replicates. AM fungal communities were distinguished by higher relative abundances of the unclassified fungal group and taxa belonging to Basidiobolaceae.

### 3.3. Phylogenetic Distribution and Functional Prediction of Bacterial and Fungal Communities

**Figure 4:**
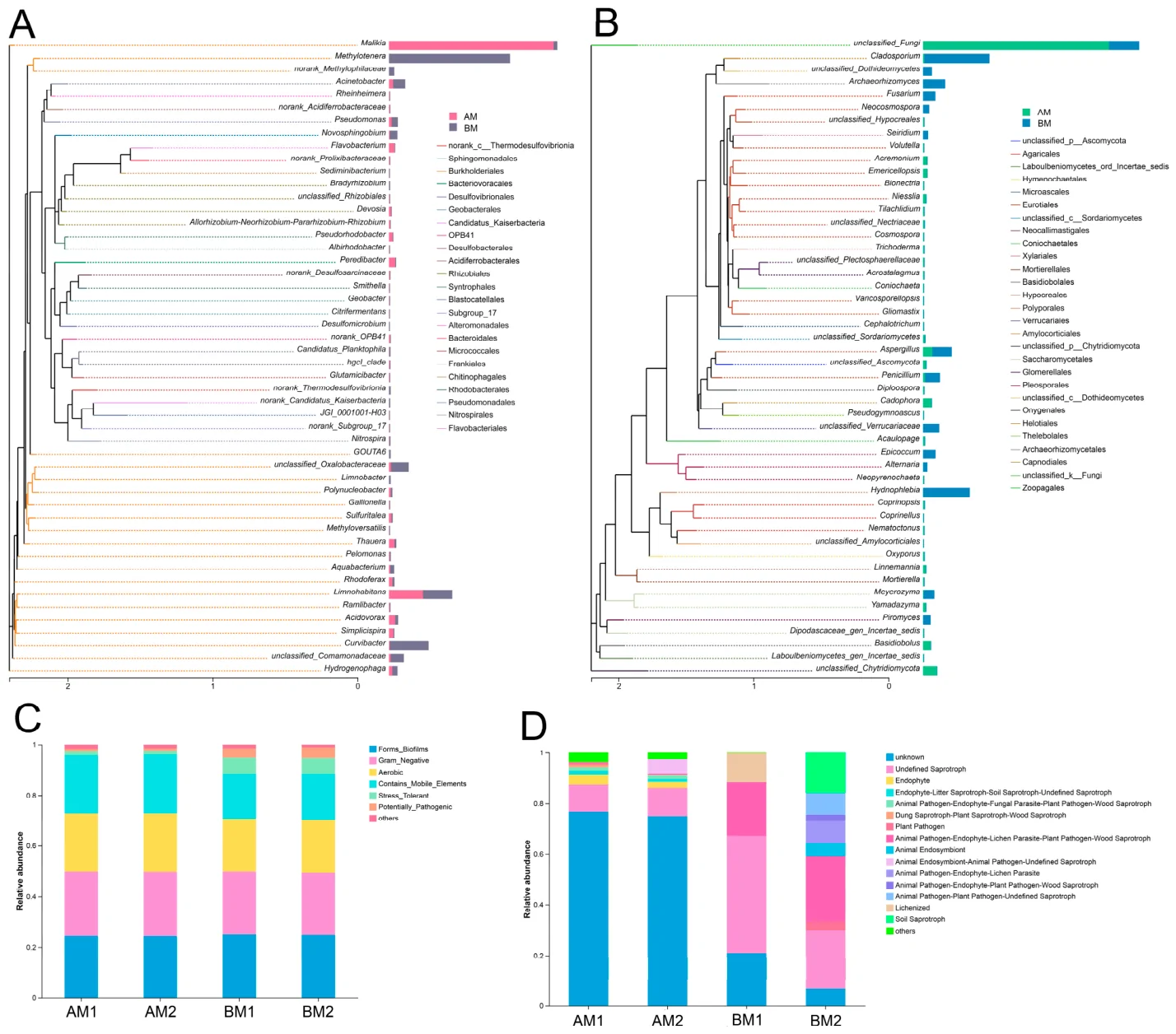
Phylogenetic distribution of bacterial (**A**) and fungal (**B**) taxa using linear discriminant analysis, and predicted functional profiles of bacterial phenotypes using BugBase (**C**) and fungal trophic modes using FUNGuild (**D**) in AM and BM samples.

Relative microbial community composition and predicted functional characteristics of the water samples are presented in Figure 4. Compositional divergence between AM and BM samples was observed for bacterial and fungal communities based on PERMANOVA analysis with a False Discovery Rate adjusted *p* < 0.05. Community differences were characterized by changes in relative abundances of specific taxa, including *Cladosporium*. Phenotypic potentials were assigned to approximately 75 percent of the total bacterial sequences via the BugBase algorithm. Relative abundances of bacterial taxa assigned phenotypic potentials related to biofilm formation and aerobic metabolism remained similar between groups. These functional prediction profiles represent the annotated subset of the microbiome rather than confirmed metabolic activities. FUNGuild assignments differed between sampling periods. Relative proportions of all fungal functional guilds were calculated utilizing the total number of quality filtered sequences per sample as the denominator. Pre-mining samples contained a high proportion of functionally unassigned sequences and higher taxonomic clarity at the genus level. Post-mining samples were dominated by a fungal group unclassified at the genus level with functional assignments based on broader familial taxonomic affiliations. Higher relative proportions of taxa computationally assigned to potential plant pathogen and animal pathogen guilds were observed in post-mining samples. Predicted trophic potentials rather than verified pathogenic functions within the localized environment are indicated by these algorithmic assignments.

### 3.4. Relationships Between Environmental Factors and Microbial Communities

**Figure 5:**
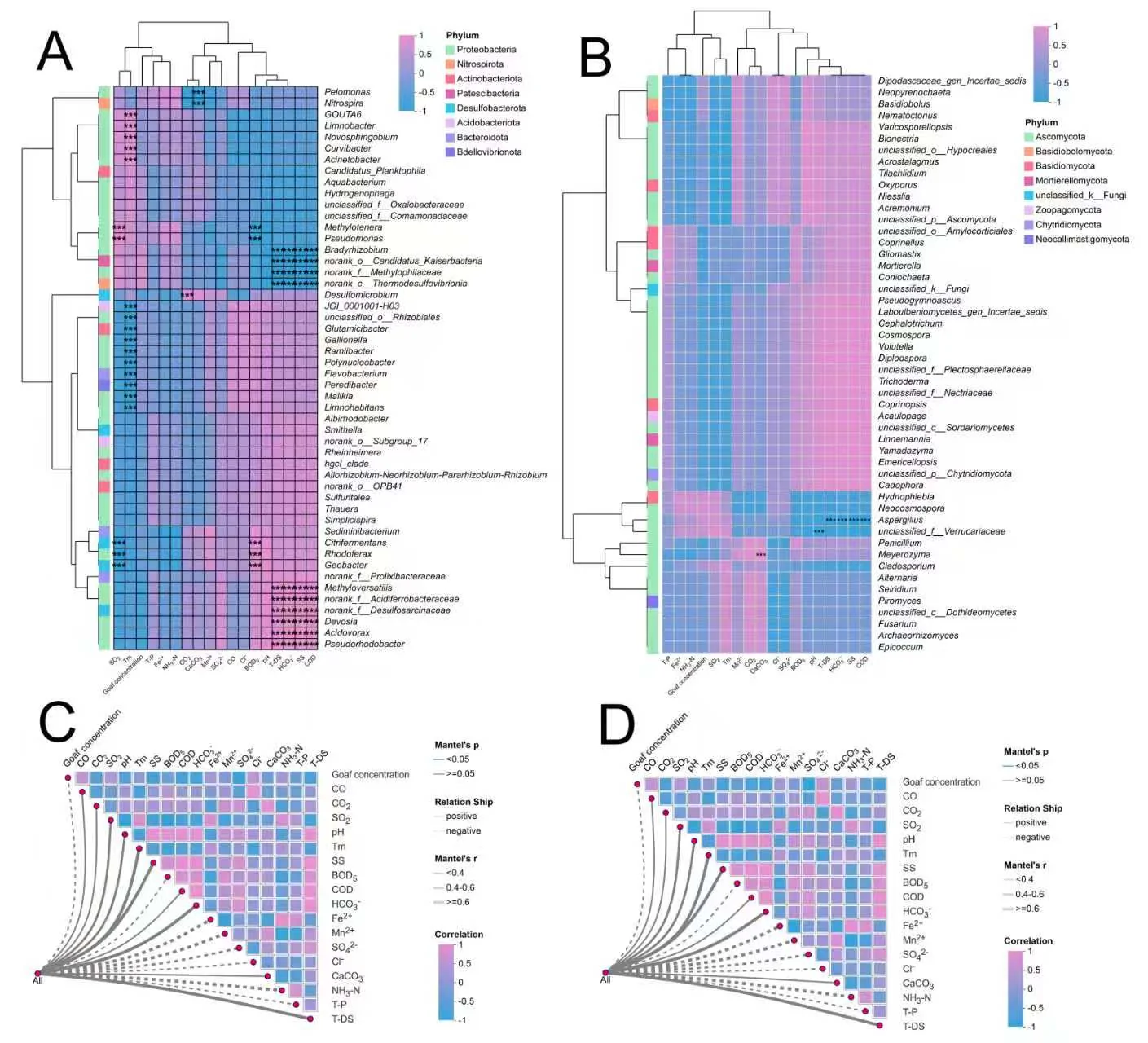
Spearman correlation heatmaps of the top bacterial (**A**) and fungal (**B**) genera with environmental variables, and Mantel test analyses of the correlations between environmental factors and bacterial (**C**) and fungal (**D**) community structures. *** indicates *p* < 0.001 (highly significant).

Statistical associations between environmental variables and community composition were evaluated using Mantel tests and Spearman correlation analyses (Figure 5). Genera, including *Devosia*, *Acidovorax*, and *Pseudorhodobacter*, in the bacterial community (Figure 5A) were positively correlated with pH, COD, and T-DS (*r* = X, *p* < Y) and negatively correlated with goaf related gas indicators (CO, CO_2_) (*r* = X, *p* < Y) and SO_2_ (*r* = X, *p* < Y). *Aspergillus* and *Penicillium* in the fungal community (Figure 5B) were positively correlated with pH and T-DS (*r* = X, *p* < Y). Shifts in water chemistry covaried with relative abundances of specific fungal taxa based on these mathematical relationships. Organic loading indicators (BOD_5_, COD) and dissolved gases were statistically linked with overall assembly patterns of bacterial and fungal communities based on network analyses (Figure 5C,D). These findings represent exploratory associations rather than definitive causal relationships given the three independent biological replicates per sampling condition. Alterations in chemical, physical, and organic parameters represent associated factors in aquatic microbial restructuring rather than primary drivers.

### 3.5. Structural Equation Models Illustrating the Environmental Drivers of Microbial Communities

**Figure 6:**
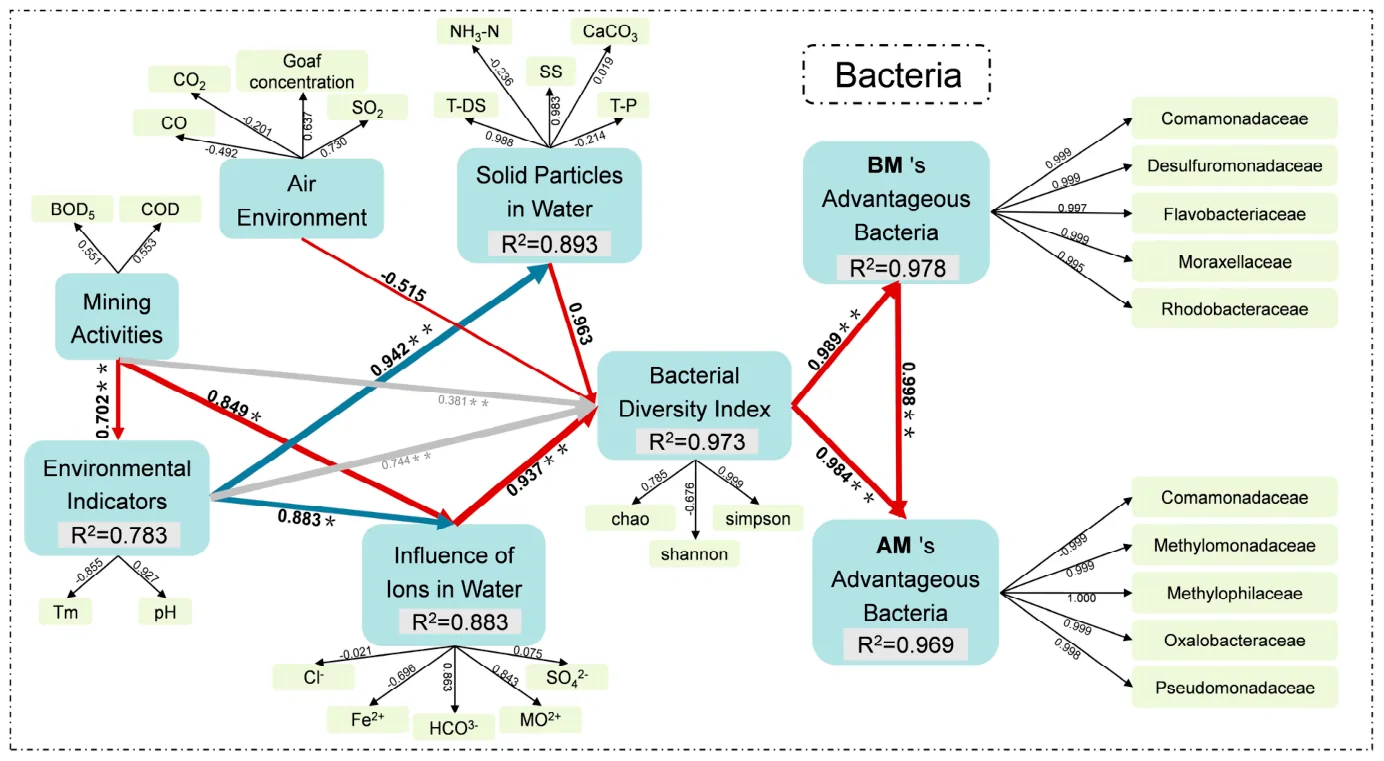
Structural equation model revealing the pathways through which mining activities and environmental factors drive bacterial community assembly in goaf water. * indicates *p* < 0.05 (significant), ** indicates *p* < 0.01 (very significant).

**Figure 7:**
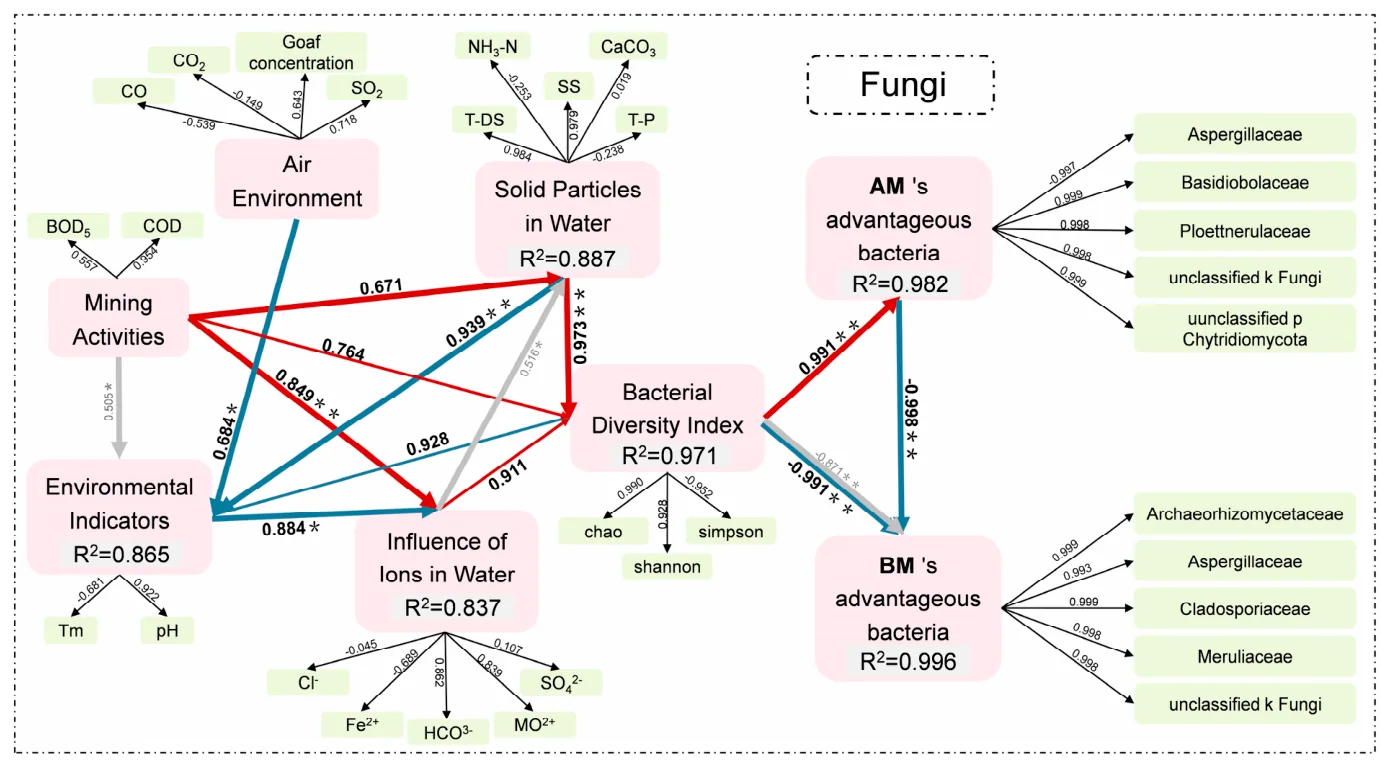
Structural equation model revealing the pathways through which mining activities and environmental factors drive fungal community assembly in goaf water. * indicates *p* < 0.05 (significant), ** indicates *p* < 0.01 (very significant).

Regarding the bacterial community (Figure 6), mining activities exhibited strong positive associations with environmental indicator scores (path coefficient 0.702, *p* < X) and water ion accumulation (path coefficient = 0.849, *p* < X). These environmental variables collectively accounted for a substantial proportion of the variance in the bacterial diversity index (*R*^2^ = 0.973). Solid particle accumulation (path coefficient = 0.963, *p* < X) and ion concentration (path coefficient = 0.937, *p* < X) showed positive statistical linkages with bacterial diversity, whereas the air environment presented a negative association (path coefficient = −0.515, *p* < X). We observed that the diversity patterns covaried with the compositional differences between the periods, marked by the transition from pre-mining prominent families, including Comamonadaceae and Desulfuromonadaceae (*R*^2^ = 0.978), to post-mining prominent families, including Methylomonadaceae and Pseudomonadaceae (*R*^2^ = 0.969). Analysis of the parallel structural equation model for the fungal community (Figure 7) demonstrated that mining activities were positively associated with solid particle (path coefficient = 0.671, *p* < X) and ion concentrations (path coefficient = 0.849, *p* < X). These physicochemical variations were closely linked with fungal diversity (*R*^2^ = 0.971). The latent variables representing the respective community characteristics showed a strong positive correlation with the AM period (path coefficient = 0.991, *p* < X) and a negative correlation with the BM period (path coefficient = −0.991, *p* < X). Within this analytical framework, taxa including Basidiobolaceae, Ploettnerulaceae, and Chytridiomycota served as mathematical indicators measuring the latent construct of the post-mining fungal community (*R*^2^ = 0.982). These structural equation models represent exploratory pathways of statistical associations rather than causal relationships due to sample size limitations and the absence of continuous time series data. Data from these models indicate that physicochemical alterations induced by mining are associated with microbial community assemblies rather than acting as deterministic drivers.

## 4. Discussion

Statistical associations between aquatic microbial community characteristics and physicochemical measurements in the Talahao coal mine during two sampling periods were evaluated. Differences in microbial community composition and lower relative diversity indices were associated with the AM period. The second sampling period covaried with higher relative proportions of taxa previously reported in disturbed environments. Variations in water physicochemical properties correlated with shifts in community structure. These relationships indicate that physicochemical differences correspond to altered microbial assembly patterns. Specific microbial community features serve as candidate indicators for assessing environmental disturbance. Developing biological monitoring tools requires validation through independent regional sampling and established continuous baselines to separate mining impacts from seasonal or hydrological variations. Current findings align with environmental filtering principles observed in regional metal mine studies, although alkaline and high particulate conditions at Talahao present aquatic selective pressures [24].

Differences in relative taxonomic compositions of bacterial and fungal communities were identified between the two sampling periods. Compositional data from amplicon sequencing sum to unity. The relative increase of specific taxa, including *Malikia*, may reflect a decline in absolute cellular abundance of other community members rather than biological proliferation. Shifts in relative proportions are consistent with statistical models of environmental filtering. The AM samples contained a high relative proportion of fungal sequences lacking genus level classification. This taxonomic ambiguity reflects limited representation of subterranean aquatic lineages within the internal transcribed spacer reference database rather than biological emergence of rare taxa. The unclassified category encompasses diverse lineages and does not constitute a single ecologically homogeneous group. Treating this aggregate category as a single dominant community introduces methodological bias. Differences in predicted fungal guild compositions suggest modifications in substrate utilization. These compositional shifts alter carbon mineralization rates and enzymatic activities during site recovery [25]. Future studies measuring absolute biogeochemical fluxes are required to test this prediction. Similar microbial diversity reductions are reported in open pit coal mining, yet the high proportion of unclassified fungi at the Talahao underground site indicates a subterranean response pattern uncaptured by surface soil reference frameworks [26,27].

Statistical linkages between hydrochemical parameters and relative abundances of microbial taxa were identified. The correlation between pH and community variation aligns with the biological reliance of microbial enzymatic activity on hydrogen ion concentrations [28,29]. The positive association between pH and genera, including *Devosia* and *Acidovorax*, reflects that alkaline conditions of the mine water fall within optimal growth ranges for these chemoheterotrophs [30,31,32,33,34,35]. These taxa possess genetic pathways for degrading complex organics and provide a biological basis for covariance with chemical oxygen demand. The influx of exogenous carbon substrates homogenizes spatial resources and favors opportunistic heterotrophs over specialized oligotrophs [36,37]. Increased organic loading enables a limited number of competitive microorganisms to dominate available ecological niches and contributes to the decline in overall microbial diversity [38]. Elevated total dissolved solids and suspended particles introduce physical and osmotic challenges. High ionic strength requires cellular osmoregulatory mechanisms. Taxa lacking these physiological adaptations experience competitive exclusion. This physical stressor provides a biological mechanism for the reduced community evenness in the compositional data [39,40]. Evaluating absolute quantitative changes across a broader temporal scale is required to isolate the causal impact of mining from background hydrological fluctuations. Mining introduces suspended particles and organic matter into aquatic systems and increases carbon substrate availability [41]. Elevated organic loading homogenizes spatial resource distributions. Resource homogenization facilitates the dominance of opportunistic taxa and concomitant reductions in alpha diversity under resource competition models [42]. Elevated total dissolved solids and ion concentrations alter water chemistry. Changes in osmotic and ionic conditions impose physiological stress. Community composition reflects the differential tolerance of resident taxa [43,44].

Indirect pathways linking mining to microbial communities were identified via structural equation modeling. Mining was positively associated with ion accumulation and suspended particle concentrations. These factors covaried with bacterial diversity and fungal community composition. The absence of a direct path from mining to microbial communities supports a model where physicochemical changes mediate mining effects [45]. This pattern aligns with the environmental filtering framework where disturbance alters habitat conditions to select for tolerant taxa. Environmental indicators, including pH and temperature, covaried with ion concentration and solid particle accumulation based on structural equation models. Bacterial diversity and fungal community composition were correlated in the models. This correlation reflects shared environmental responses rather than direct biotic interactions [46]. Bacteria and fungi participate in nutrient cycling. Alterations in bacterial community structure modify substrate availability for fungi. This mechanism remains hypothetical without experimental evidence.

Bacterial inferred functional profiles remained stable following taxonomic restructuring based on functional predictions. This stability aligns with the functional redundancy hypothesis where multiple taxa perform equivalent ecological functions to maintain biogeochemical processes. Distinguishing functional redundancy from phylogenetically conserved core metabolism requires an analysis of functional and taxonomic decoupling using shotgun metagenomics. Fungal trophic guilds exhibited higher compositional variability than bacterial functional profiles. This discrepancy reflects differences in bacterial and fungal responses to environmental change. Whether this represents functional plasticity in fungi or methodological differences in prediction approaches remains unresolved. Concurrent analysis of bacterial and fungal communities provides perspectives on ecosystem function given these groups mediate distinct interconnected biogeochemical processes [47].

Coal mining alters aquatic microbial community composition and diversity through physicochemical changes in mine water. Covariation between environmental variables and microbial communities supports the application of microbial monitoring as an assessment tool. Indirect pathways affecting microbial communities and differential responses between bacterial and fungal functional stability were identified by integrating bacterial and fungal analyses with structural equation modeling.

## 5. Conclusions

This study identified differences in aquatic microbial communities between the pre-mining and post-mining periods at the Talahao coal mine. The second sampling period coincided with altered physicochemical water properties, lower microbial community evenness, and distinct taxonomic compositions across both bacterial and fungal domains. The compositional data demonstrate the relative enrichment of specific taxa, including the bacterial genus *Malikia* and unclassified fungal lineages, following mining operations. Exploratory structural equation modeling indicates that these physicochemical variations are statistically associated with the observed microbial restructuring. Computational predictions suggest divergent phenotypic potentials between the two domains, with bacterial profiles showing less compositional variation than the predicted fungal trophic assignments. Assessing both bacterial and fungal communities concurrently offers valuable biological candidate indicators for monitoring environmental associations within subterranean aquatic ecosystems.

## Figures and Tables

**Table 1:** Monitoring data table of gas component concentration in goaf.

	Goaf CH_4_ Concentration %	CO µg·g^−1^	CO_2_%	H_2_S µg·g^−1^	SO_2_ µg·g^−1^
AM 1-1	0.15	7.1	0.27	\	0.52
AM 1-2	0.22	6.3	0.31	\	0.12
AM 1-3	0.13	8.1	0.45	\	0.87
AM 2-1	0.19	9.3	0.23	\	0.34
AM 2-2	0.24	6	0.38	\	0.67
AM 2-3	0.17	7.8	0.42	\	0.23
BM 1-1	0.28	5.8	0.29	\	0.76
BM 1-2	0.18	8.6	0.35	\	0.43
BM 1-3	0.2	8.2	0.26	\	0.98
BM 2-1	0.21	5.9	0.44	\	0.55
BM 2-2	0.26	6.8	0.33	\	0.59
BM 2-3	0.12	6.8	0.41	\	0.71

**Table 2:** Monitoring data table of physical and chemical indicators of water quality in goaf.

		AM 1-1	AM 1-2	AM 1-3	AM 2-1	AM 2-2	AM 2-3	BM 1-1	BM 1-2	BM 1-3	BM 2-1	BM 2-2	BM 2-3
pH	\	7.2	6.8	7.1	6.8	7.2	7.1	4.7	4.7	4.5	5.3	5.6	5.4
Tm	°C	18.7	19.5	21.3	17.4	20.8	16.9	22.3	19.7	24.8	20.5	25.1	21.4
SS	mg·L^−1^	427	389	465	412	371	441	89	95	81	98	79	101
BOD_5_		11	7	15	9	16	13	11	9	10	12	9	10
COD		206	247	221	197	235	227	127	95	149	164	189	201
HCO^3^^−^		174	159	188	165	182	153	31	27	45	38	24	42
Fe^2+^		67.4	53.1	78.9	45.6	34.2	44.8	81.5	79.1	68.5	46.8	60.9	53.7
Mn^2+^		1.2	1.3	1.3	1.7	1.5	1.4	0.8	0.9	0.9	1.1	1.6	1.3
SO_4_^2−^		2789	2345	3120	2654	2218	2976	2533	2891	2177	3045	2412	2728
CL^−^		67	91	53	105	78	94	112	85	79	45	73	68
CaCO_3_		412	587	445	509	391	618	476	529	399	546	491	601
NH_3_-N		11.6	9.3	13.7	8.8	14.2	10.5	12.8	7.9	15.3	11.1	9.8	13.2
T-P		1.7	2.1	2.5	1.4	2.3	1.9	2.6	1.6	2	2.4	1.5	2.2
T-DS		1187	1024	1456	1333	998	1271	157	203	134	249	188	121

Note: Abbreviations denote specific sample groups and physicochemical water quality parameters evaluated in Table 2. SS indicates suspended solids. BOD_5_ refers to five-day biochemical oxygen demand. COD stands for chemical oxygen demand. HCO^3−^ represents bicarbonate concentration. Fe^2+^ indicates ferrous iron. Mn^2+^ represents manganese. SO_4_^2−^ denotes sulfate. CL^−^ stands for chloride. CaCO_3_ represents calcium carbonate. NH_3_-N indicates ammonia nitrogen. T-P refers to total phosphorus. T-DS stands for total dissolved solids.

## Data Availability

The original contributions presented in this study are included in the article. Further inquiries can be directed to the corresponding authors.
